# Fitness Consequences of Timing of Migration and Breeding in Cormorants

**DOI:** 10.1371/journal.pone.0046165

**Published:** 2012-09-25

**Authors:** Phillip Gienapp, Thomas Bregnballe

**Affiliations:** 1 Department of Biosciences, University of Helsinki, Helsinki, Finland; 2 Department of Bioscience, Aarhus University, Rønde, Denmark; Institute of Ecology, Germany

## Abstract

In most bird species timing of breeding affects reproductive success whereby early breeding is favoured. In migratory species migration time, especially arrival at the breeding grounds, and breeding time are expected to be correlated. Consequently, migration time should also have fitness consequences. However, in contrast to breeding time, evidence for fitness consequences of migration time is much more limited. Climate change has been shown to negatively affect the synchrony between trophic levels thereby leading to directional selection on timing but again direct evidence in avian migration time is scarce. We here analysed fitness consequences of migration and breeding time in great cormorants and tested whether climate change has led to increased selection on timing using a long-term data set from a breeding colony on the island of Vorsø (Denmark). Reproductive success, measured as number of fledglings, correlated with breeding time and arrival time at the colony and declined during the season. This seasonal decline became steeper during the study period for both migration and breeding time and was positively correlated to winter/spring climate, i.e. selection was stronger after warmer winters/springs. However, the increasing selection pressure on timing seems to be unrelated to climate change as the climatic variables that were related to selection strength did not increase during the study period. There is indirect evidence that phenology or abundances of preferred prey species have changed which could have altered selection on timing of migration and breeding.

## Introduction

In seasonal environments the timing of life-cycle events generally has strong consequences for reproductive success or survival as environmental conditions (e.g. food supply), are favourable only for a limited period. For example, on the one hand migrating birds should not arrive too early at their breeding grounds because harsh environmental conditions, such as cold spells, may pose a mortality risk [1]. On the other hand, individuals that arrive later at the breeding grounds may face stronger competition by conspecifics and may have difficulties finding a suitable mate or breeding territory [2]–[4].

While a number of studies reported a relationship between breeding time and reproductive success in birds, e.g.[5], [6]–[8], the evidence in migration time is more limited. Bety *et al*. [9] tracked radio-tagged snow geese and found that reproductive success increased and then declined with arrival time in females with an optimal arrival time of three days before median arrival. A similar relationship has been found between departure date from the last staging area and reproductive success in barnacle geese [10]. In black kites early arriving individuals had higher reproductive success, partly because they were able to settle in high quality territories [3]. Evidence is also mounting in songbirds that earlier arriving individuals have a higher reproduce success [4], [11]. The fact that there are much fewer studies on avian migration time than breeding time is likely due to methodological limitations. Monitoring breeding attempts and their timing is simple in many species, especially in cavity breeders accepting nest boxes, because it only requires locating the nest and monitoring breeding phenology and success. To quantify fitness consequences of migration time it is however necessary to record individual migration time and link it to the following breeding attempt. Furthermore, accurately recording individual migration time has been difficult in many species, e.g. woodland living passerines.

**Figure 1:**
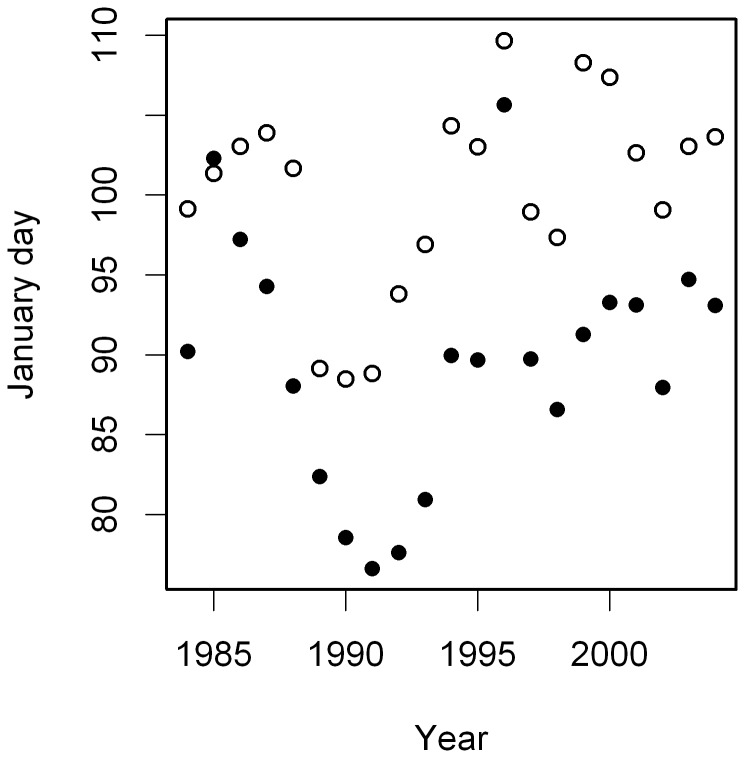
Temporal trends in timing of migration and breeding during the study period. Mean arrival date at the breeding colony (filled symbols) and mean laying date (open symbols) plotted against year.

Climate change has disrupted the synchrony between predator-prey relationships in a number of ecological networks. For example, rising spring temperatures have advanced the phenology of caterpillars, an important prey items for insectivorous birds, more strongly than the breeding phenology of the birds, which has led to strong selection for earlier breeding [12], [13]. Similar temporal mismatches through non-linear or temporally heterogeneous climate change have been reported for other predator-prey systems, e.g. in plankton species [14], shellfish and shrimp [15] or seabirds and fish [16]. While it has been inferred that such selection on breeding time has also led to selection on migration time in flycatchers [17] so far no study has reported direct evidence for climate change induced selection on migration time. It has however been shown that migratory bird species did not seem to fully track temperature changes in their breeding areas – possibly indicating a mismatch with food phenology – and that species that were able to better track temperature trends showed less population declines [18], [19].

Cormorants are short-distance migrants and nest colonially, often in trees. Individuals generally appear in the colony shortly after arrival on the breeding grounds [20]. Colour-ringing of chicks and subsequent daily resighting of adults in the Vorsø colony (Denmark) made it possible to record individual arrival dates at the colony and reproductive success. Here we here use this data set to analyse the relationship between arrival time at the colony and breeding time, fitness consequences of arrival and breeding time and changes in these relationships over the last two decades.

**Figure 2:**
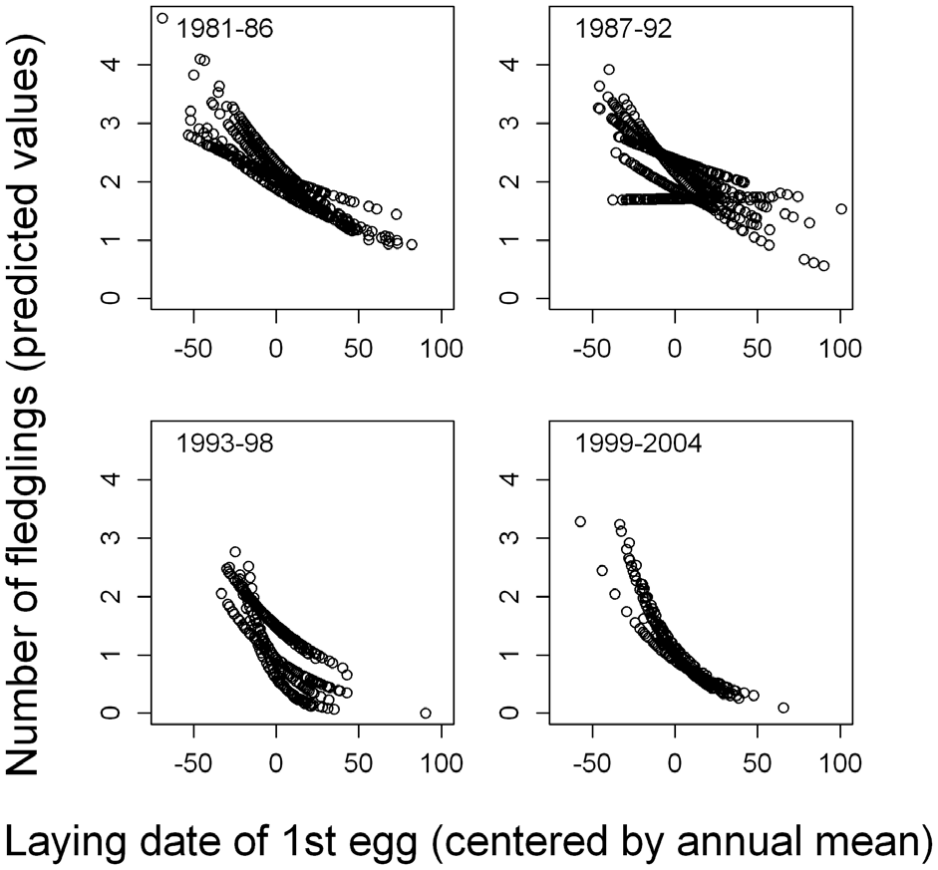
Relationship between breeding time and annual reproductive success. The number of fledged chicks (predicted values from model) is plotted against egg laying date, separately for each year. Reproductive success declined during the breeding season. This decline differed among years and became stronger during the study period (see text for statistical details). For illustrative purposes the years were combined into four periods and displayed separately.

**Table 1:** Analyses of annual reproductive success, measured as the number of fledged chicks, in relation to breeding (a) and arrival time (b) and both (c).

	Var.	*b*	se	*t*	df	*p*
a)						
female	4.4e−21					
laying date		−0.008	0.001	−5.83	319	<0.001
year		−0.056	0.006	−9.41	319	<0.001
age		0.116	0.029	3.96	319	<0.001
age^2^		−0.007	0.002	−3.40	319	<0.001
b)						
female	0					
arrival date		−0.003	0.001	−2.53	292	0.011
year		−0.064	0.006	−10.2	292	<0.001
age		0.121	0.030	4.03	292	<0.001
age^2^		−0.007	0.002	−3.25	292	0.001
male	4.1e−16					
arrival date		−0.003	0.001	−2.85	317	0.005
year		−0.064	0.006	−11.08	317	<0.001
age		0.139	0.032	4.39	317	<0.001
age^2^		−0.008	0.002	−3.75	317	<0.001
c)						
female	0					
arrival date		0.0003	0.0012	0.27	291	0.79
laying date		−0.008	0.001	−5.24	292	<0.001
year		−0.061	0.006	−9.61	292	<0.001
age		0.103	0.030	3.41	292	<0.001
age^2^		−0.006	0.002	−2.85	292	0.005
male	2.8e−12					
arrival date		0.0007	0.0011	0.68	316	0.50
laying date		−0.011	0.001	−7.53	317	<0.001
year		−0.062	0.006	−10.8	317	<0.001
age		0.115	0.0318	3.61	317	<0.001
age^2^		−0.007	0.002	−3.17	317	0.002

Results of the GLMM (Poisson-distribution and log link-function) with the explained variance by the random effect (Var.), the parameter estimate for fixed effects (*b*), its standard error (se), *t* calculated from *b* and se, denominator degrees of freedom (df) and significance of t-test (*p*).

## Methods

### Study Species

The great cormorant (*Phalacrocorax carbo*) is a large, long lived, fish eating colonial waterbird. The largest of the two subspecies occurring in Europe mainly breeds along exposed coasts in UK and Norway, whereas the other subspecies (*P. c. sinensis*) mainly breeds on the continent near to more sheltered coasts or near to lagoons and lakes. The largest breeding populations in Europe are found around the Baltic Sea and the Black Sea but other countries rich in coasts and/or lakes, like The Netherlands, also constitutes important breeding areas [21]. The populations on the continent were very small until they became protected during the 1970s, after which numbers increased by a factor of 30 within 30 years [22], [23]. The *sinensis* subspecies which this study concerns breeds in small or large colonies holding up to 13,000 pairs [21]. The birds start to breed at the age of 2–4 years usually with a new partner each year, and up to 2–4 chicks are raised to fledging when food conditions are favourable near to the colony [24]–[26]. Breeders tend to forage within 20 km of the colony but they fly up to 60 km during periods and years with reduced food availability [27]. The length of the foraging trips has a direct influence on the number of daily feedings for chicks and thereby on fledgling production. The vast majority of cormorants breeding in the Baltic Sea region migrate south and southwest in autumn with individuals from the same colony widely dispersing over Europe south to the coast of North Africa for the winter [20]. Individuals tend to return to the same wintering areas each year [28], but birds wintering in the central and more northern parts of Europe move further south and west in cold winters. The distance that adults in the present study colony have to migrate in spring range from less than 200 km up to 2500 km [29].

**Figure 3:**
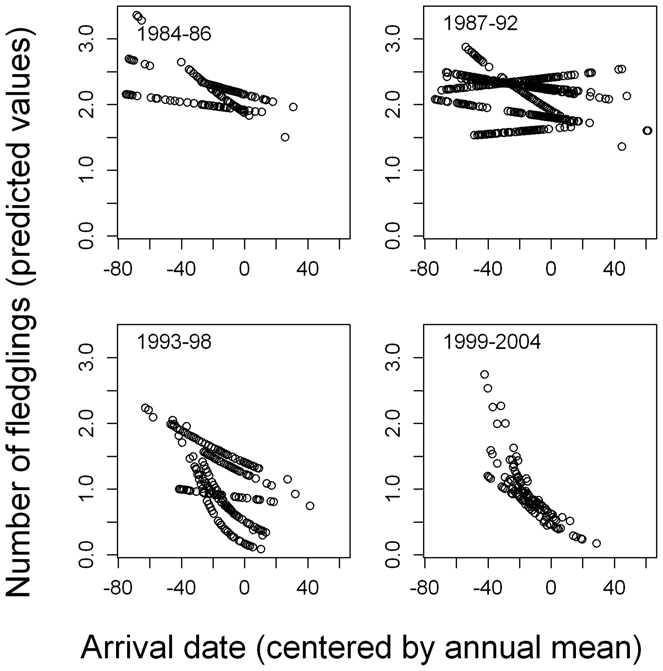
Relationship between arrival time at the colony and annual reproductive success. The number of fledged chicks (predicted values from model) is plotted against arrival time. separately by year. The number of fledged chicks declined with arrival time. This decline differed among years and became stronger during the study period (see text for statistical details). For illustrative purposes the years were combined into four periods and displayed separately. Only data for males shown.

**Table 2:** Selection differentials (S) and standardised selection differentials (*i*) on breeding and arrival time.

	breeding time			arrival time		
year	S	i	n	S	i	n
1984	−2.54	−0.12	210	1.14	0.04	209
1985	−2.88	−0.18	211	−1.71	−0.09	226
1986	−2.54	−0.18	334	−3.99	−0.21	348
1987	−3.12	−0.20	307	−2.70	−0.17	344
1988	−2.36	−0.15	343	−2.61	−0.12	330
1989	−1.16	−0,07	255	−0,22	−0.01	316
1990	−2.73	−0.14	335	−1.78	−0.07	339
1991	−1.60	−0.11	233	−2.69	−0.10	334
1992	0.14	0.01	137	0.07	0.00	233
1993	−3.26	−0.23	239	−3.20	−0.15	251
1994	−2.09	−0.20	161	−4.29	−0.30	253
1995	−5.17	−0.45	209	−10.23	−0.68	267
1996	−4.56	−0.45	212	−4.62	−0.47	140
1997	−7.07	−0.59	220	−8.05	−0.61	158
1998	−6.72	−0.38	232	−1.41	−0.07	124
1999	−5.79	−0.36	238	−3.22	−0.24	77
2000	−5.70	−0.44	367	−7.06	−0.47	99
2001	−5.08	−0.40	252	−9.05	−0.66	81
2002	−5.97	−0.42	32	−5.32	−0.36	60
2003	−3.56	−0.31	17	−2.63	−0.29	42
2004	−2.04	−0.27	278	−5.37	−0.43	79
mean	−3.61	−0.27		−3.76	−0.26	

*n* gives the sample size, i.e. number of breeding pairs (‘breeding time’) and number of individuals (‘arrival time’) for which reproductive success was recorded.

### Study Area and Long-term Monitoring

The study was carried out in a tree-nesting great cormorant (*P. c. sinensis*) colony located in the nature reserve on the island of Vorsø (Denmark, 55.87° N, 10.17°E). The first cormorants bred on Vorsø in 1944. Until 1970 cormorant numbers were regulated by shooting. After hunting control stopped the number of breeding pairs increased from around 250 pairs to 5000 pairs in the early 1990s and then decreased to 530 in 2011.

**Figure 4:**
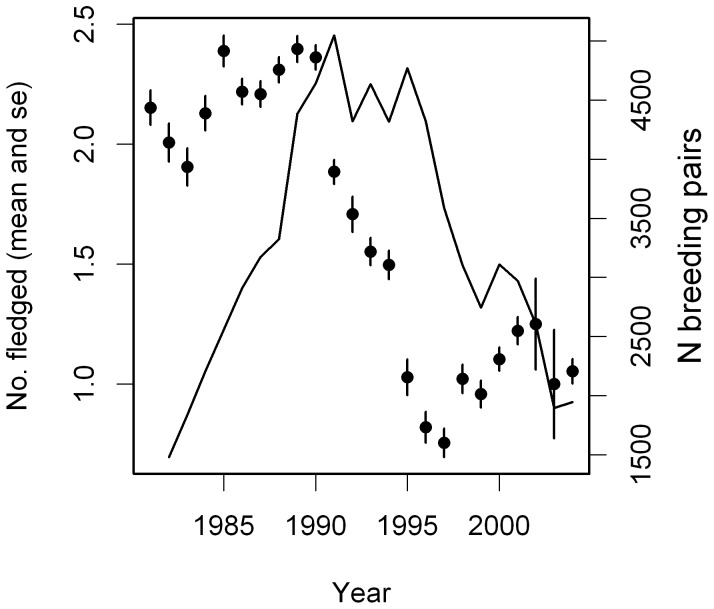
Changes in reproductive success and population numbers during the study period. Mean ± se of annual reproductive success at the Vorsø colony (filled circles), and number of breeding pairs (solid line) for the period 1981 until 2004.

From 1977 onwards 440 chicks per year were ringed on average in the colony with uniquely coded colour-rings to facilitate monitoring of individuals in the colony. From 1983 the colony was visited daily to read rings and record breeding attempts. From 1984 onwards observations were made from an 8 m high tower located at the rim of the colony. Access to the tower was possible through a covered walkway which allowed the observers to enter and leave the tower without disturbing the birds. Observations of colour-ringed birds were made one to three times for a total of one to four hours per day. Since monitoring took place throughout the period when birds were present in the colony, i.e. from January (in mild winters) until October, the total number of observation hours amounted to 500–800 hours per year. Based on the daily searches for colour-ringed birds and records of breeding activity, it was possible to monitor time of arrival at the colony, the laying date of the first egg of a clutch (‘laying date’), and reproductive success. Regular monitoring of the colony, including daily observations and ringing of chicks, ended in 2004.

Between 1981 and 2004 the date of the first egg and annual reproductive success (ARS), measured as the number of fledged chicks, were recorded for a total of 5775 breeding attempts. However, the identity of individuals was not known in all cases since not all adult cormorants in the colony had colour-rings. The number of monitored breeding attempts per year varied between 17 in 2003 and 367 in 2000 with an average of about 250.

**Table 3:** Prey choice of cormorants at the Vorsø colony.

	February–April		May–July	
	1980–83	1993–94	1980–83	1993–94
bull rout(*Myoxocephalus scorpius*)	39 (31–57)	5 (0–13)	17 (10–28)	2 (0–8)
eelpout(*Zoarces viviparous*)	15 (0–29)	8 (0–16)	24 (7–45)	4 (0–18)
dab(*Limanda limanda*)	21 (0–39)	47 (23–74)	29 (7–55)	53 (48–57)
other saltwater species^1^	25 (10–40)	38 (28–49)	30 (20–40)	36 (23–52)
freshwater species	0	4 (0–9)	2 (0–8)	7 (0–22)

Mean (range) percentage of weight of caught fish species during pre-laying and incubation (Feb–Apr) and chick-rearing (May–Jul). Percentage of weight per species in prey was determined from otoliths in pellets collected at the colony.

1 including eel (*Anguilla anguilla*) and three-spined stickleback (*Gasterosteus aculeatus*).

Since detection probability and accuracy of arrival dates increased after the construction of the observation tower, the analysis of arrival time was restricted to years from 1984 onwards. In this period, in total 17531 arrival dates and 5775 laying dates were recorded. Reproductive success was recorded for all these 5775 laying dates but for only 4310 out of these 17531 arrival dates. However, arrival date, laying date and annual reproductive success were recorded for only 569 colour-ringed males and 581 colour-ringed females totalling 1525 and 1385 observations, respectively.

Permission to work at the Vorsø nature reserve was provided by the Danish Ministry of Environment. Permission to ring the chicks was granted by the Ringing Centre at the Zoological Museum of Copenhagen.

**Table 4:** Relationship between selection on breeding time, measured as slope of GLM, and year, climatic variable and population density, measured as number of breeding pairs.

rank	AIC	*b*	se	*F*_1,12_	*b*	se	*F*_1,12_	*b*	se	*F*_1,11_
1	−93.11	−0.002	0.0004	19.9***	0.010	0.002	14.1**	−1.2e−6	2.8e−6	0.21 ^n.s.^
2	−89.99	−0.001	0.0005	7.90*	0.005	0.002	9.18*	−3.5e−6	3.1e−6	1.30 ^n.s.^
3	−87.30	−0.002	0.0005	18.1**	0.006	0.002	5.70*	−6.2e−7	3.4e−6	0.03 ^n.s.^
4	−86.05	−0.002	0.005	13.3**	0.004	0.002	4.19†	−3.6e−6	3.6e−6	1.00 ^n.s.^
5	−85.46	−0.002	0.005	9.31*	0.006	0.003	3.66†	−1.4e−6	3.6e−6	0.14 ^n.s.^

The five best models are presented here and the climatic variables included in the model were: 1) mean January water temperature, 2) mean January air temperature, 3) mean May air temperature, 4) March-May NAO index and 5) mean May water temperature.

n.s. *p*>0.28,

† *p*<0.10,

* *p*<0.05,

** *p*<0.01,

*** p<0.001.

### Analysis of Prey Use

Cormorants regurgitate indigestible food items when roosting. These regurgitated pellets were collected in the colony and manually searched for otoliths. From the otoliths the consumed fish species were identified. The length and weight of the caught fish were calculated from the otoliths’ length by using species specific equations [30], [31]. Pellets were collected during a period from February until July in 1980–83 (16–136 pellets per month) and in 1993–94 (29–45 pellets per month).

### Climate Data

Monthly mean air temperature for December until May was calculated from mean daily air temperature recorded at the weather station of the Danish Meteorological Institute that was closest to the study area (Billund airport 55.73° N, 9.1°E, distance to study area: 68 km). Monthly mean water temperature was calculated from mean daily water temperature at two stations of the Danish Meteorological Institute, Rørvig (55.57° N, 11.46°E, distance to study area: 85 km, years: 1984, 1989–1995, 1997/8) and København (55.41° N, 12.36°E, distance to study area: 145 km, years: 2000–2004). Unfortunately, we were unable to obtain data sets on water temperature that covered longer and overlapping periods. Data on seasonal North Atlantic Oscillation (NAO) [32] indices were obtained from the website of the National Center for Atmospheric Research (http://www.cgd.ucar.edu/cas/jhurrell/indices.html). The following seasonal indices were analysed: December–February, January–March, February–April and March–May.

### Statistical Analyses

Annual reproductive success (ARS) was analysed using generalised linear mixed models (GLMM) with Poisson-distributions and log link-functions. In these models individuals with only a single record (247 males and 284 females) were excluded because including them would affect the precision with which the individual variance component could be estimated [33]. When we found that individual as random effect explained no variation, generalised linear models (GLM) with Poisson-distributions and log link-functions were further used. This increased the breeding time-data set to 5775 records because also unringed individuals with known laying date and reproductive success could be included. Since laying date is under female ‘control’ [34] we included only female identity as a random effect when analysing fitness consequences of breeding time.

Statistical significance of fixed effects in mixed models was assessed using *t*-tests based on parameter estimates and their standard error. The corresponding denominator df were calculated using the number of individuals as total df. This is a conservative approach and sample sizes were so large that alternative calculations of df would not affect significance levels [33]. Minimum adequate models were obtained by stepwise deletion of non-significant variables from full models, starting with interactions.

The correlation between arrival and breeding time was estimated by fitting a Bayesian bivariate mixed model with both arrival and breeding time as dependent variables, age as fixed effect and individual as random effect using an informative prior. The correlation between arrival and breeding time at the individual level was then calculated from the covariance and variances. Statistical significance was assessed by comparing the Deviance Information Criterion (DIC) of this model to the DIC of a model where the covariance between arrival and breeding time was constrained to zero.

Since climatic variables tend to show high correlations (average of pairwise correlations of all variables = 0.36) and such high collinearity in independent variables is problematic, we ran separate models for each climatic variable and selected the best model based on model fit. Year and population density, measured as the number of breeding pairs in the colony, were included as additional explanatory variables in each separate analyses.

Selection differentials and standardised selection differentials were calculated following the standard approach [35].

All analyses were carried out in R 2.14.1 [36] using the packages ‘lme4’ and ‘MCMCglmm’.

## Results

Timing of migration, i.e. arrival at the colony, and timing of breeding did not show significant temporal trends during the study period (arrival date: *b* = 0.12±0.28, *F*_1,19_ = 0.18, *p* = 0.68; laying date: *b* = 0.33±0.21, *F*_1,19_ = 2.44, *p* = 0.14) (Fig. 1). The interval between mean arrival and mean egg laying did not change, either (*b* = 0.21±0.15, *F*_1,19_ = 1.94, *p* = 0.18).

Arrival time at the colony and breeding time, i.e. the date when the first egg of the clutch was laid, were positively correlated in both males and females (males: *r* = 0.85, 95% CI = 0.74–0.96, ΔDIC = 104.8; females: *r* = 0.82, 95% CI = 0.71–0.88, ΔDIC = 135.1).

Annual reproductive success (ARS), measured as the number of chicks fledged, was related to breeding time, year and age (Table 1a). ARS declined with laying date (Fig. 2) and decreased during the study period. There was evidence for senescence in reproductive success, even after controlling for breeding time, as the quadratic effect of age was negative and statistically significant (Table 1a).

Since a breeding pair has only one laying date but two arrival dates, fitness consequences of arrival time were tested separately for each sex. As expected from the decline in ARS and the correlation of breeding and arrival time with breeding time ARS also declined with arrival time in both males and females (Table 1b). Furthermore, as in the analysis of breeding time, ARS decreased over the study period and there were significant negative quadratic relationships with age, for both males and females (Table 1b). When laying date was included as explanatory variable, arrival time was rendered non-significant in both females and males (Table 1c).

In all analyses of fitness consequences of breeding and arrival time individual identity explained no variation (Table 1a, b, c) which means analysing ARS with generalised linear models was valid.

Selection on breeding time, i.e. the seasonal decline in reproductive success with breeding time, became stronger over time (interaction laying date*year, *b* = −0.001±0.0001, *Χ* ^2^ = 79.7, df = 1, *p*<0.001, GLM) (Fig. 2). As in breeding time, selection on arrival time became stronger during the study period (interaction arrival date*year, females: *b* = −0.0006±0.0003, *Χ* ^2^ = 4.40, df = 1, *p* = 0.036, males: b = −0.0012±0.0003, *Χ* ^2^ = 20.1, df = 1, *p<*0.001, GLM) (Fig. 3). The mean selection differential for breeding time was −3.6 days (s.e. = 0.43) and for arrival time −3.8 days (s.e. = 0.65), while the respective standardised selection differentials were −0.27 (s.e. = 0.03) and −0.26 (s.e. = 0.05) (Table 2).

Mean ARS declined significantly during the study period (*b* = −0.068±0.010, *F*_1,22_ = 50.5, p<0.001). As can be seen in Fig. 4 reproductive success seemed to be stable until 1990 with on average 2.21±0.05 chicks fledged per breeding pair, from 1991 to 1994 it declined steeply, and after that seemed to level off with only 1.02±0.05 fledged chicks per pair on average. Colony-wide reproductive success was not related to the number of breeding pairs at the Vorsø colony (*F*_1,21_ = 0.011, *p* = 0.92). However, when we tested for effects of delayed density dependence we found that reproductive success was not affected by breeding density in the previous year (*F*_1,20_ = 0.92, p = 0.35) but with a delay of two (b = −0.0003±0.0001, *F*_1,19_ = 5.73, p = 0.027) and three years (−0.0003±0.0001, *F*_1,18_ = 12.1, p = 0.003), respectively.

The proportion of prey species changed slightly during the season but more strongly between years (Table 3). In the 1980s bull rout was the most important prey species during pre-laying and incubation (Feb–Apr) but was less often caught late during the chick-rearing period (May–Jul), while the proportions of the other species changed little. In the 1990s the within-season variation was also small but much more strikingly the proportion of bull rout and eelpout taken both early and late in the season dropped from on average 25% to only 5%.

Monthly mean air temperatures increased by 0.10 to 0.15°C per year but only significantly so in April (*b* = 0.10±0.03, *F*_1,19_ = 11.5, *p* = 0.003). Monthly mean water temperatures increased significantly by 0.17°C per year in December (*F*_1,13_ = 13.0, *p* = 0.003) while they changed only very little (−0.05 to 0.01°C per year) in the months January to May.

There was evidence that the intensity of selection on breeding time, quantified as the slope from the GLM, increased with ambient temperature. In the best five models the seasonal decline in reproductive success was positively correlated with January water temperature, January air temperature, May air temperature, May water temperature or March-May NAO index (Table 4). However, these variables did not fully explain the increasing selection as year was significant in each model. Population density did not affect the strength of selection (Table 4).

## Discussion

Although it is generally assumed that timing of migration in birds is important for individual fitness, documented evidence for this is relatively scarce [37]. We found here that annual reproductive success, measured as the number of fledged chicks, indeed declined with arrival time in the studied cormorant colony (Fig. 3). Arrival time at the colony and breeding time were positively correlated and the fitness consequences of arrival time could be explained by this close link because reproductive success declined during the breeding season, which is a common pattern in birds, e.g. [6], [38], [39].

There is one general problem with the measurement and interpretation of phenotypic selection [40]: the observed relationship between fitness and trait, here timing of migration and breeding, could possibly be caused by a third, unmeasured, variable that affects fitness and trait simultaneously. In the context of avian breeding time this problem is generally discussed as the distinction between the ‘timing hypothesis’ e.g. [7], [8] versus the ‘quality hypothesis’,e.g. [8], [41]. While under the first a true causal relationship between timing and reproductive success exists, under the second individuals differ in ‘quality’ and individuals of ‘good quality’ are able to raise many offspring and to breed early. The main problem here is to identify and measure ‘quality’, i.e. the variable being possibly responsible for the correlation between fitness and trait. Consequently, rather than trying to include this variable in the selection analysis, it is more realistic to address this issue by experimentally manipulating breeding time [6] or by comparing phenotypic with genotypic selection [40] and so far the evidence for avian breeding time points to a causal relationship between timing of breeding and reproductive success [6], [7], [42]–[44].

We also found that the selection on arrival and breeding time has increased during the study period (Figs. 2 and 3). Climate change has been shown to have advanced the prey phenology in several predator-prey systems [45], [46] and if the predator fails to track this advancement this mis-match can lead to increased selection for a phenological advancement in the predator [e.g. 13]. Arrival and breeding time showed no advancement in the cormorants (Fig. 2). This pattern would hence be consistent with climate change creating a phenological mis-match between predator and prey.

Cormorants prey almost entirely on fish e.g,. [47], [48], [49] and it has been shown that prey abundance strongly affects reproductive success in cormorants and other seabirds [50]–[55]. The abundance of a relevant prey species can easily change during the breeding season, especially in open waters where certain fish species migrate in or out of the shallow areas, which are preferred hunting areas of the cormorants [24]. Climate change may have affected this seasonal pattern of prey abundance by advancing the appearance and disappearance of certain species in the cormorants’ hunting areas. Increasing temperatures may not only affect the phenology [56]–[58] but also the life-history and thereby directly the abundance of fish available as prey [59].

Because no data on fish phenology in the cormorants’ preferred hunting areas are available, we could only indirectly test whether increasing temperatures have intensified selection on timing. To test this we regressed selection intensity, measured as the slope from the corresponding GLM, against temperature and NAO and found that the strength of selection was positively correlated to winter and spring temperatures. There was no evidence that population density, as measured by the number of breeding pairs in the colony, would affect the strength of selection. While temperatures increased as expected from climate change [60] this trend was statistically non-significant for the climatic variables that correlated with the strength of selection. With 15 years the available data set for water temperatures was however not very large and it may hence have been a power problem to detect significant trends in climatic variables. Furthermore, these temperatures explained only part of the increase in selection as year was significant in all models.

While no data on fish abundance was available, we could analyse relative abundance of fish species that the studied cormorants caught from regurgitated pellets. The relative prey abundance changed only little over the season but strikingly over time (Table 3). This does not necessarily imply that absolute abundances did not change as the analysis of pellets only indicates choice or relative abundances but not absolute abundances. We however think that the most parsimonious explanation would be a change in prey abundance rather than a change in preference of the cormorants, which are generalist predators [48], [49].

In conclusion, we found that – as expected but rarely reported – migration time affects reproductive success because early arriving individuals are able to breed early and reproductive success declines during the breeding season. The seasonal decline in reproductive success was positively correlated with winter and spring climate, i.e. selection was stronger after warmer winters and springs. Selection also became stronger during the study period. This increasing selection could not be fully explained, however, by warming temperatures. The relative abundance of prey species taken by the cormorants changed during the study period, which likely indicates a change in abundance of these species. It hence seems that a change in prey abundance, whether driven by climate change or not, would have led to the increased selection on breeding time.

Consistent selection on a heritable trait should lead to an evolutionary response. Currently, we do not know whether timing of breeding and migration are heritable in cormorants but several aspects of migratory behaviour have been shown to be heritable reviewed in [61], [62] and to respond to selection [63], [64]. Timing of breeding also generally shows moderate heritability in birds [43], [44], [65], [66]. Consequently, we might expect an evolutionary response to selection, which would however be slow due to the long generation time of cormorants.
